# Bis[5-(2-pyrid­yl)pyrazine-2-carbonitrile]­silver(I) tetra­fluorido­borate

**DOI:** 10.1107/S1600536810035178

**Published:** 2010-09-08

**Authors:** Zi-Jia Wang, Fan Zhang, Chong-Qing Wan

**Affiliations:** a Department of Chemistry, Capital Normal University, Beijing 100048, People’s Republic of China

## Abstract

In the title mononuclear complex, [Ag(C_10_H_6_N_4_)_2_]BF_4_, the Ag^I^ atom adopts a square-planar N_4 _coordination geometry and is surrounded by two 5-(2-pyrid­yl)pyrazine-2-carbonitrile ligands. The tetra­fluorido­borate anions link the mononuclear cations through inter­molecular C—H⋯F hydrogen-bonding inter­actions, forming an infinite tape structure along [110]. Other weak inter­actions occur: π–π stacking with centroid–centroid distances of 3.820 (2) and 3.898 (1) Å between pyridyl rings and 3.610 (2) and 3.926 (2) Å between pyrazinyl rings as well as F⋯π contacts involving the tetra­fluorido­borate anions and pyrazine rings [F⋯centroid = 2.999 (3) Å]; these combine with the hydrogen-bonding inter­actions to link the mononuclear cations into a three-dimensional supra­molecular architecture.

## Related literature

For coordination complexes with 2,2′-bipyridine, see: Casini *et al.* (2006 ▶); Li *et al.* (2010 ▶); Wang *et al.* (2009 ▶). For other related structures involving 2,2′-bipyridine derivatives, see: Berghian *et al.* (2005 ▶); Mathieu *et al.* (2001 ▶). For the coordination chemistry of multidentate N-containing ligands, see: Peedikakkal & Vittal (2010 ▶). For properties of pyridine-based ligands, see: Casini *et al.* (2006 ▶). For comparison Ag—N(pyrazin­yl) distances, see: Biju & Rajasekharan (2008 ▶). For C—H⋯F inter­actions, see: Denis *et al.* (2004 ▶). For a comparable BF_4_ anion–pyrazinyl inter­action, see: Wan *et al.* (2008 ▶).
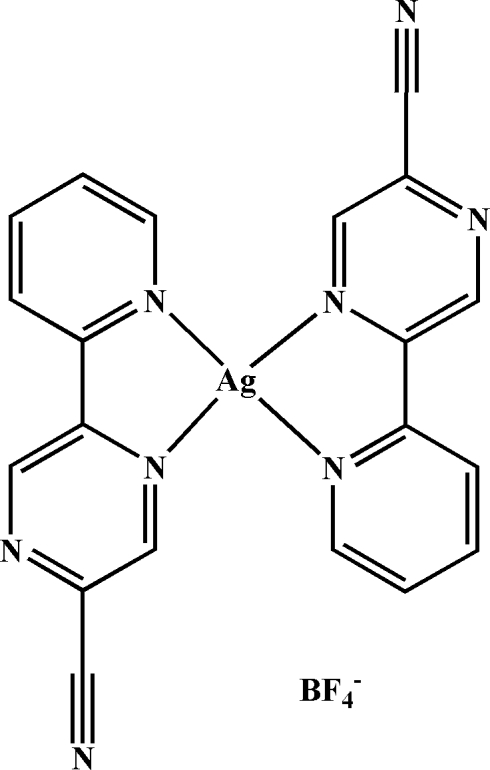


## Experimental

### 

#### Crystal data

[Ag(C_10_H_6_N_4_)_2_]BF_4_
*M* *_r_* = 559.06
Triclinic, 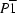

*a* = 7.8144 (12) Å
*b* = 11.2492 (16) Å
*c* = 12.2697 (18) Å
α = 104.168 (3)°
β = 90.789 (2)°
γ = 101.429 (3)°
*V* = 1022.8 (3) Å^3^
*Z* = 2
Mo *K*α radiation
μ = 1.05 mm^−1^
*T* = 293 K
0.45 × 0.40 × 0.30 mm

#### Data collection

Bruker APEXII CCD area-detector diffractometer
Absorption correction: multi-scan (*SADABS*; Bruker, 2007 ▶) *T*_min_ = 0.615, *T*_max_ = 0.783
7178 measured reflections
4986 independent reflections
3929 reflections with *I* > 2σ(*I*)
*R*_int_ = 0.017

#### Refinement

*R*[*F* ^2^ > 2σ(*F* ^2^)] = 0.038
*wR*(*F* ^2^) = 0.111
*S* = 1.04
4986 reflections
307 parameters
H-atom parameters constrained
Δρ_max_ = 0.95 e Å^−3^
Δρ_min_ = −0.73 e Å^−3^

### 

Data collection: *APEX2* (Bruker, 2007 ▶); cell refinement: *APEX2* and *SAINT* (Bruker, 2007 ▶); data reduction: *SAINT*; program(s) used to solve structure: *SHELXS97* (Sheldrick, 2008 ▶); program(s) used to refine structure: *SHELXL97* (Sheldrick, 2008 ▶); molecular graphics: *SHELXTL* (Sheldrick, 2008 ▶); software used to prepare material for publication: *SHELXTL* and *PLATON* (Spek, 2009 ▶).

## Supplementary Material

Crystal structure: contains datablocks I, global. DOI: 10.1107/S1600536810035178/bg2359sup1.cif

Structure factors: contains datablocks I. DOI: 10.1107/S1600536810035178/bg2359Isup2.hkl

Additional supplementary materials:  crystallographic information; 3D view; checkCIF report

## Figures and Tables

**Table 1:** Hydrogen-bond geometry (Å, °)

*D*—H⋯*A*	*D*—H	H⋯*A*	*D*⋯*A*	*D*—H⋯*A*
C5—H5a⋯F3^i^	0.93	2.49	3.398 (3)	167
C13—H13a⋯F2	0.93	2.43	3.015 (5)	121
C11—H11a⋯F4^ii^	0.93	2.39	3.132 (4)	137

Symmetry codes: (i) 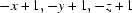; (ii) 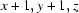.

**Fig. 1.**
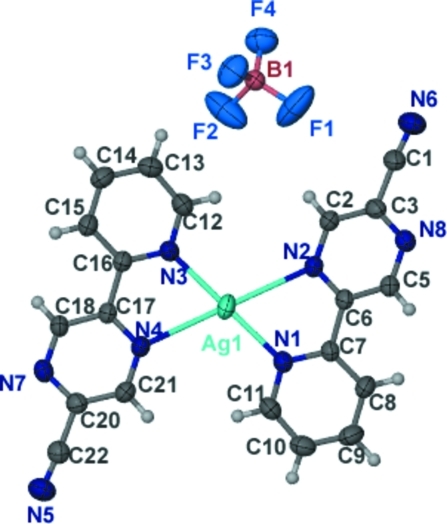
The atom-numbering scheme of the title [Ag(C10H6N4)2]BF4. Displacement ellipsoids are drawn at the 50% probability level and H atoms are shown as sticks of arbitrary radii.

**Fig. 2.**
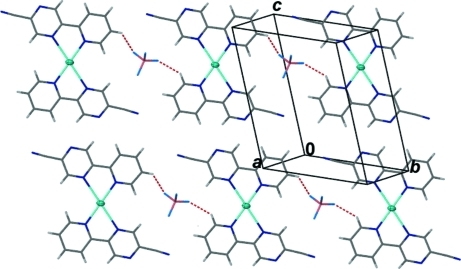
The tetrafluoridoborate linkages between the [Ag(C10H6N4)2]+moieties arranged along [110] direction. The red-dashed lines indicate the C—H···F hydrogen-bonding interactions. Symmetry code: i x-1, y-1, z.

**Fig. 3.**
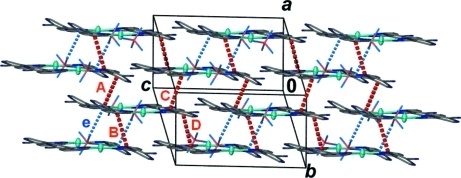
Three-dimensional structure of the title complex. The red dashed lines represent π–π stacking interactions,while blue dashed lines indicate F(BF4-)···π(pyrazinyl) interactions. A, B, C and D red lettering indicate Cg2···Cg2i,Cg3···Cg3ii,Cg4···Cg4iii and Cg1···Cg1iv distances, respectively, while E represents F4···Cg1v distance. (See Table 2).

## supplementary crystallographic information

### Comment

During the past decades, coordination chemistry based on multidentate N-containing ligands has been widely developed and received intense interests (Peedikakkal *et al.*, 2010). 2,2'-bipyridine is a popular member of the pyridine-based family and attracts a great of attentions because of the potential medicinal applications (Casini *et al.*, 2006) and the fascinating framework structures (Li *et al.*, 2010; Wang *et al.*, 2009) of its divers metal complexes. Many 2,2'-bipyridine derivatives together with their various metal complexes have also been synthesized and well characterized (Berghian *et al.*, 2005; Mathieu *et al.*, 2001).

Herein, we present the structure of the new complex [Ag(C_10_H_6_N_4_)_2_]BF_4_ derived from 5-(2-pyridyl)-2-cyanopyrazine ligand, a similar ligand to the 2,2'-bipyridine featuring a 2-cyanopyrazinyl group at the 2-pyridyl carbon atom (Scheme 1). In the mononuclear title complex, the two ligands surrounding the center Ag(I) ion are in an anti-relationship and almost in the same plane, thus each of them chelates the Ag(I) ion through one 2-pyridyl N atom and one 4-pyrazinyl N atom, leading to a square planar *N4*- coordination geometry. As shown in Fig.1, the Ag1—N1(pyridyl) and Ag1—N3(pyridyl) bonds equal to 2.196 (2) and 2.203 (2) Å, respectively, which are considerably shorter than the other two Ag—N(pyrazinyl) bonds with the distances of 2.659 (2) Å (Ag1—N4) and 2.685 (2) Å (Ag1—N2), respectively. The longer Ag—N(pyrazinyl) distance is comparable to that in [Ag(dafone)_2_]NO_3_.H_2_O (dafone = 4,5-diazafluoren-9-one) (Biju *et al.*, 2008). For the tetrafluoridoborate anions, each one links two neighbor [Ag(C_10_H_6_N_4_)_2_]^+^ cationic moieties arranged along the [110] direction through C—H···F (Denis *et al.*, 2004) hydrogen bonding (C13···F2 3.015 (1) Å, C13—H13a···F2 120.8°; C11^i^···F4 3.132 (2) Å, C11^i^—H11a^i^···F4 137.2°, i *x*-1, *y*-1, *z*), forming an infinite tape structure (Fig. 2). The tape arrays are arranged along [110] direction in a shoulder to shoulder mode and stack along [-110] direction via π-π and F(BF_4_^-^)···π(pyrazinyl) interactions (Fig. 3). The close centroid(pyridyl)···centroid(pyridyl)distances are 3.820 (2) and 3.898 (1) Å, while that of centroid(pyrazinyl)···centroid(pyrazinyl) are 3.610 (2) and 3.926 (2) Å. For the anion-π interaction, F4(BF_4_^-^)···centroid(pyrazinyl) distance is 2.999 (3) Å, comparable to that 3.097 (1) Å found in Cu[(2-C_4_H_3_N_2_)_2_C(OH)_2_]_2_(BF_4_^-^)_2_ (Wan, *et al.*, 2008).

### Experimental

The ligand 5-(2-pyridyl)-2-cyanopyrazine was obtained commercially.The ligand (0.182 g, 0.1 mmol) was dissolved in a mixture of methanol, 2 ml, and acetonitrile, 2 ml was added to AgBF_4_ (0.194g, 0.1mmol), with constantly stirring. After four hours, the clear solution was filtered and kept in air for one week at room temperature to yield colorless rod-like crystals (274 mg, 72% yeild).

### Refinement

The hydrogen atoms were placed in idealized positions and allowed to ride on the relevant carbon atoms, with C— H = 0.93 Å and *U*ĩso~(H) = 1.2Ueq(C).

### Crystal data

**Table:**

[Ag(C_10_H_6_N_4_)_2_]BF_4_	*Z* = 2
*M**_r_* = 559.06	*F*(000) = 552
Triclinic, *P*1	*D*_x_ = 1.815 Mg m^−^^3^
Hall symbol: -P 1	Mo *K*α radiation, λ = 0.71073 Å
*a* = 7.8144 (12) Å	Cell parameters from 255 reflections
*b* = 11.2492 (16) Å	θ = 1.9–28.2°
*c* = 12.2697 (18) Å	µ = 1.05 mm^−^^1^
α = 104.168 (3)°	*T* = 293 K
β = 90.789 (2)°	Rod, colourless
γ = 101.429 (3)°	0.45 × 0.40 × 0.30 mm
*V* = 1022.8 (3) Å^3^	

### Data collection

**Table:**

Bruker APEXII CCD area-detector diffractometer	4986 independent reflections
Radiation source: fine-focus sealed tube	3929 reflections with *I* > 2σ(*I*)
graphite	*R*_int_ = 0.017
ω scans	θ_max_ = 28.3°, θ_min_ = 1.9°
Absorption correction: multi-scan (*SADABS*; Bruker, 2007)	*h* = −10→10
*T*_min_ = 0.615, *T*_max_ = 0.783	*k* = −15→11
7178 measured reflections	*l* = −11→16

### Refinement

**Table:**

Refinement on *F*^2^	Primary atom site location: structure-invariant direct methods
Least-squares matrix: full	Secondary atom site location: difference Fourier map
*R*[*F*^2^ > 2σ(*F*^2^)] = 0.038	Hydrogen site location: inferred from neighbouring sites
*wR*(*F*^2^) = 0.111	H-atom parameters constrained
*S* = 1.04	*w* = 1/[σ^2^(*F*_o_^2^) + (0.0606*P*)^2^ + 0.3679*P*] where *P* = (*F*_o_^2^ + 2*F*_c_^2^)/3
4986 reflections	(Δ/σ)_max_ < 0.001
307 parameters	Δρ_max_ = 0.95 e Å^−^^3^
0 restraints	Δρ_min_ = −0.73 e Å^−^^3^

### Special details

**Table:**

Geometry. All esds (except the esd in the dihedral angle between two l.s. planes) are estimated using the full covariance matrix. The cell esds are taken into account individually in the estimation of esds in distances, angles and torsion angles; correlations between esds in cell parameters are only used when they are defined by crystal symmetry. An approximate (isotropic) treatment of cell esds is used for estimating esds involving l.s. planes.
Refinement. Refinement of F^2^ against ALL reflections. The weighted R-factor wR and goodness of fit S are based on F^2^, conventional R-factors R are based on F, with F set to zero for negative F^2^. The threshold expression of F^2^ > 2sigma(F^2^) is used only for calculating R-factors(gt) etc. and is not relevant to the choice of reflections for refinement. R-factors based on F^2^ are statistically about twice as large as those based on F, and R- factors based on ALL data will be even larger.

### Fractional atomic coordinates and isotropic or equivalent isotropic displacement parameters (Å^2^)

**Table:**

	*x*	*y*	*z*	*U*_iso_*/*U*_eq_	
Ag1	0.48906 (4)	0.75499 (2)	0.295313 (17)	0.05808 (11)	
N1	0.6368 (3)	0.8859 (2)	0.44508 (18)	0.0413 (5)	
C17	0.4863 (3)	0.7511 (2)	0.0256 (2)	0.0354 (5)	
C8	0.7174 (4)	0.9522 (3)	0.6418 (2)	0.0488 (7)	
H8A	0.7096	0.9362	0.7126	0.059*	
C9	0.8161 (4)	1.0650 (3)	0.6289 (3)	0.0536 (7)	
H9A	0.8757	1.1251	0.6908	0.064*	
N3	0.3487 (3)	0.6200 (2)	0.14425 (19)	0.0440 (5)	
N7	0.5870 (3)	0.8837 (2)	−0.09560 (19)	0.0448 (5)	
C6	0.5258 (3)	0.7428 (2)	0.5588 (2)	0.0349 (5)	
C13	0.1720 (4)	0.4170 (3)	0.0664 (3)	0.0552 (7)	
H13A	0.1052	0.3437	0.0784	0.066*	
C7	0.6308 (3)	0.8640 (2)	0.5475 (2)	0.0366 (5)	
C10	0.8236 (4)	1.0857 (3)	0.5235 (3)	0.0525 (7)	
H10A	0.8897	1.1596	0.5123	0.063*	
C16	0.3722 (3)	0.6342 (2)	0.0397 (2)	0.0366 (5)	
C3	0.3250 (4)	0.5376 (2)	0.5951 (2)	0.0396 (5)	
C18	0.4773 (4)	0.7863 (3)	−0.0758 (2)	0.0437 (6)	
H18A	0.3912	0.7396	−0.1314	0.052*	
C11	0.7318 (4)	0.9954 (3)	0.4349 (3)	0.0507 (7)	
H11A	0.7356	1.0108	0.3638	0.061*	
C20	0.7024 (3)	0.9492 (2)	−0.0119 (2)	0.0403 (5)	
C14	0.1954 (4)	0.4306 (3)	−0.0405 (3)	0.0511 (7)	
H14A	0.1437	0.3670	−0.1025	0.061*	
N5	0.9098 (4)	1.1363 (3)	−0.0604 (3)	0.0645 (7)	
C22	0.8224 (4)	1.0544 (3)	−0.0359 (2)	0.0481 (6)	
C21	0.7073 (4)	0.9214 (3)	0.0921 (2)	0.0475 (6)	
H21A	0.7873	0.9725	0.1496	0.057*	
C1	0.2107 (4)	0.4315 (3)	0.6209 (2)	0.0509 (7)	
C15	0.2969 (4)	0.5401 (3)	−0.0547 (2)	0.0458 (6)	
H15A	0.3150	0.5511	−0.1266	0.055*	
C12	0.2491 (4)	0.5138 (3)	0.1556 (3)	0.0552 (7)	
H12A	0.2307	0.5046	0.2281	0.066*	
N6	0.1246 (5)	0.3511 (3)	0.6475 (3)	0.0784 (10)	
F3	0.0390 (3)	0.1799 (2)	0.2096 (2)	0.0787 (6)	
B1	−0.0554 (4)	0.2501 (3)	0.2781 (3)	0.0411 (6)	
F1	0.0294 (4)	0.3082 (4)	0.3781 (3)	0.1278 (13)	
F2	−0.0835 (4)	0.3504 (3)	0.2347 (3)	0.1227 (12)	
F4	−0.2181 (3)	0.1894 (2)	0.2873 (2)	0.0778 (6)	
N4	0.5982 (3)	0.8218 (2)	0.11044 (18)	0.0445 (5)	
N8	0.4633 (3)	0.5922 (2)	0.66795 (19)	0.0418 (5)	
C5	0.5636 (3)	0.6942 (2)	0.6495 (2)	0.0387 (5)	
H5A	0.6617	0.7348	0.6980	0.046*	
N2	0.3941 (3)	0.6827 (2)	0.48248 (18)	0.0402 (5)	
C2	0.2927 (4)	0.5793 (2)	0.5009 (2)	0.0420 (6)	
H2B	0.1992	0.5350	0.4498	0.050*	

### Atomic displacement parameters (Å^2^)

**Table:**

	*U*^11^	*U*^22^	*U*^33^	*U*^12^	*U*^13^	*U*^23^
Ag1	0.0852 (2)	0.05274 (16)	0.02856 (13)	0.00162 (12)	−0.00852 (11)	0.00651 (9)
N1	0.0523 (13)	0.0386 (11)	0.0313 (11)	0.0033 (10)	0.0040 (9)	0.0102 (9)
C17	0.0408 (13)	0.0365 (12)	0.0269 (11)	0.0062 (10)	0.0027 (9)	0.0059 (9)
C8	0.0643 (18)	0.0420 (14)	0.0336 (13)	0.0004 (13)	−0.0073 (12)	0.0064 (11)
C9	0.0606 (18)	0.0383 (14)	0.0524 (17)	−0.0031 (13)	−0.0108 (14)	0.0052 (12)
N3	0.0541 (13)	0.0412 (11)	0.0320 (11)	−0.0036 (10)	0.0019 (10)	0.0113 (9)
N7	0.0503 (13)	0.0492 (13)	0.0342 (11)	0.0025 (10)	0.0034 (10)	0.0155 (10)
C6	0.0424 (13)	0.0345 (11)	0.0263 (11)	0.0064 (10)	0.0051 (9)	0.0058 (9)
C13	0.0599 (18)	0.0445 (15)	0.0550 (18)	−0.0077 (13)	0.0015 (14)	0.0160 (13)
C7	0.0438 (13)	0.0359 (12)	0.0292 (11)	0.0054 (10)	0.0034 (10)	0.0089 (9)
C10	0.0558 (17)	0.0394 (14)	0.0601 (19)	−0.0012 (12)	0.0014 (14)	0.0176 (13)
C16	0.0418 (13)	0.0360 (12)	0.0305 (12)	0.0056 (10)	−0.0014 (10)	0.0079 (9)
C3	0.0489 (14)	0.0314 (11)	0.0354 (13)	0.0036 (10)	0.0089 (11)	0.0058 (10)
C18	0.0504 (15)	0.0468 (14)	0.0297 (12)	−0.0019 (12)	−0.0009 (11)	0.0116 (11)
C11	0.0628 (18)	0.0465 (15)	0.0433 (15)	0.0012 (13)	0.0054 (13)	0.0204 (12)
C20	0.0447 (14)	0.0376 (12)	0.0369 (13)	0.0053 (11)	0.0063 (11)	0.0086 (10)
C14	0.0587 (17)	0.0411 (14)	0.0456 (16)	−0.0001 (12)	−0.0081 (13)	0.0051 (12)
N5	0.0714 (18)	0.0531 (15)	0.0635 (18)	−0.0065 (14)	0.0085 (15)	0.0197 (14)
C22	0.0561 (17)	0.0450 (15)	0.0409 (15)	0.0056 (13)	0.0049 (13)	0.0104 (12)
C21	0.0573 (17)	0.0427 (14)	0.0341 (13)	−0.0036 (12)	−0.0031 (12)	0.0056 (11)
C1	0.0690 (19)	0.0373 (13)	0.0402 (15)	−0.0022 (13)	0.0027 (13)	0.0092 (11)
C15	0.0596 (17)	0.0411 (13)	0.0323 (13)	0.0027 (12)	−0.0034 (12)	0.0077 (11)
C12	0.0670 (19)	0.0540 (17)	0.0401 (15)	−0.0060 (14)	0.0060 (14)	0.0180 (13)
N6	0.102 (2)	0.0543 (16)	0.065 (2)	−0.0189 (16)	0.0047 (18)	0.0184 (15)
F3	0.0780 (14)	0.0826 (14)	0.0689 (14)	0.0290 (12)	0.0006 (11)	−0.0027 (11)
B1	0.0441 (16)	0.0402 (15)	0.0358 (14)	−0.0004 (12)	0.0024 (12)	0.0110 (12)
F1	0.0818 (17)	0.168 (3)	0.084 (2)	0.0053 (18)	−0.0150 (15)	−0.0426 (19)
F2	0.143 (3)	0.101 (2)	0.159 (3)	0.0464 (19)	0.077 (2)	0.079 (2)
F4	0.0647 (12)	0.0723 (13)	0.0895 (16)	−0.0018 (10)	0.0035 (11)	0.0202 (12)
N4	0.0574 (13)	0.0404 (11)	0.0293 (10)	−0.0027 (10)	−0.0018 (10)	0.0074 (9)
N8	0.0501 (12)	0.0392 (11)	0.0364 (11)	0.0061 (10)	0.0037 (9)	0.0129 (9)
C5	0.0427 (13)	0.0403 (12)	0.0323 (12)	0.0051 (10)	0.0012 (10)	0.0103 (10)
N2	0.0486 (12)	0.0394 (11)	0.0293 (10)	0.0030 (9)	0.0003 (9)	0.0078 (9)
C2	0.0491 (15)	0.0391 (13)	0.0321 (12)	−0.0005 (11)	−0.0017 (11)	0.0066 (10)

### Geometric parameters (Å, °)

**Table:**

Ag1—N1	2.196 (2)	C16—C15	1.394 (4)
Ag1—N3	2.203 (2)	C3—N8	1.337 (4)
N1—C7	1.338 (3)	C3—C2	1.387 (4)
N1—C11	1.341 (4)	C3—C1	1.447 (4)
C17—N4	1.333 (3)	C18—H18A	0.9300
C17—C18	1.400 (3)	C11—H11A	0.9300
C17—C16	1.485 (3)	C20—C21	1.388 (4)
C8—C7	1.390 (4)	C20—C22	1.451 (4)
C8—C9	1.394 (4)	C14—C15	1.378 (4)
C8—H8A	0.9300	C14—H14A	0.9300
C9—C10	1.369 (5)	N5—C22	1.139 (4)
C9—H9A	0.9300	C21—N4	1.335 (4)
N3—C12	1.329 (4)	C21—H21A	0.9300
N3—C16	1.341 (3)	C1—N6	1.134 (4)
N7—C20	1.324 (4)	C15—H15A	0.9300
N7—C18	1.326 (4)	C12—H12A	0.9300
C6—N2	1.335 (3)	F3—B1	1.340 (4)
C6—C5	1.406 (3)	B1—F1	1.337 (4)
C6—C7	1.484 (3)	B1—F4	1.340 (4)
C13—C14	1.368 (4)	B1—F2	1.413 (4)
C13—C12	1.373 (4)	N8—C5	1.326 (3)
C13—H13A	0.9300	C5—H5A	0.9300
C10—C11	1.370 (4)	N2—C2	1.341 (3)
C10—H10A	0.9300	C2—H2B	0.9300
			
N1—Ag1—N3	177.92 (8)	C17—C18—H18A	118.8
C7—N1—C11	118.1 (2)	N1—C11—C10	123.6 (3)
C7—N1—Ag1	123.04 (17)	N1—C11—H11A	118.2
C11—N1—Ag1	118.76 (19)	C10—C11—H11A	118.2
N4—C17—C18	120.3 (2)	N7—C20—C21	122.9 (2)
N4—C17—C16	119.2 (2)	N7—C20—C22	115.0 (2)
C18—C17—C16	120.5 (2)	C21—C20—C22	122.1 (3)
C7—C8—C9	119.2 (3)	C13—C14—C15	118.9 (3)
C7—C8—H8A	120.4	C13—C14—H14A	120.6
C9—C8—H8A	120.4	C15—C14—H14A	120.6
C10—C9—C8	118.8 (3)	N5—C22—C20	175.8 (3)
C10—C9—H9A	120.6	N4—C21—C20	120.5 (2)
C8—C9—H9A	120.6	N4—C21—H21A	119.7
C12—N3—C16	118.1 (2)	C20—C21—H21A	119.7
C12—N3—Ag1	118.73 (19)	N6—C1—C3	176.1 (3)
C16—N3—Ag1	122.86 (17)	C14—C15—C16	119.4 (3)
C20—N7—C18	116.0 (2)	C14—C15—H15A	120.3
N2—C6—C5	121.1 (2)	C16—C15—H15A	120.3
N2—C6—C7	118.7 (2)	N3—C12—C13	123.7 (3)
C5—C6—C7	120.2 (2)	N3—C12—H12A	118.2
C14—C13—C12	118.6 (3)	C13—C12—H12A	118.2
C14—C13—H13A	120.7	F1—B1—F4	112.6 (3)
C12—C13—H13A	120.7	F1—B1—F3	112.4 (3)
N1—C7—C8	121.6 (2)	F4—B1—F3	114.0 (3)
N1—C7—C6	118.2 (2)	F1—B1—F2	103.0 (3)
C8—C7—C6	120.2 (2)	F4—B1—F2	103.0 (3)
C9—C10—C11	118.7 (3)	F3—B1—F2	110.8 (3)
C9—C10—H10A	120.7	C17—N4—C21	117.6 (2)
C11—C10—H10A	120.7	C5—N8—C3	116.3 (2)
N3—C16—C15	121.3 (2)	N8—C5—C6	121.8 (2)
N3—C16—C17	118.7 (2)	N8—C5—H5A	119.1
C15—C16—C17	120.0 (2)	C6—C5—H5A	119.1
N8—C3—C2	122.5 (2)	C6—N2—C2	116.9 (2)
N8—C3—C1	115.5 (2)	N2—C2—C3	121.0 (2)
C2—C3—C1	122.1 (3)	N2—C2—H2B	119.5
N7—C18—C17	122.4 (2)	C3—C2—H2B	119.5
N7—C18—H18A	118.8		
			
C7—C8—C9—C10	0.4 (5)	C9—C10—C11—N1	−1.3 (5)
C11—N1—C7—C8	1.1 (4)	C18—N7—C20—C21	−1.8 (4)
Ag1—N1—C7—C8	−176.0 (2)	C18—N7—C20—C22	179.3 (3)
C11—N1—C7—C6	−180.0 (2)	C12—C13—C14—C15	0.7 (5)
Ag1—N1—C7—C6	3.0 (3)	N7—C20—C21—N4	3.1 (4)
C9—C8—C7—N1	−1.5 (4)	C22—C20—C21—N4	−178.1 (3)
C9—C8—C7—C6	179.6 (3)	C13—C14—C15—C16	−0.3 (5)
N2—C6—C7—N1	−24.8 (3)	N3—C16—C15—C14	0.4 (4)
C5—C6—C7—N1	156.3 (2)	C17—C16—C15—C14	178.1 (3)
N2—C6—C7—C8	154.2 (3)	C16—N3—C12—C13	1.3 (5)
C5—C6—C7—C8	−24.7 (4)	Ag1—N3—C12—C13	−172.3 (3)
C8—C9—C10—C11	0.9 (5)	C14—C13—C12—N3	−1.3 (5)
C12—N3—C16—C15	−0.9 (4)	C18—C17—N4—C21	−4.3 (4)
Ag1—N3—C16—C15	172.5 (2)	C16—C17—N4—C21	175.4 (2)
C12—N3—C16—C17	−178.6 (3)	C20—C21—N4—C17	0.2 (4)
Ag1—N3—C16—C17	−5.3 (3)	C2—C3—N8—C5	4.0 (4)
N4—C17—C16—N3	18.2 (4)	C1—C3—N8—C5	−176.0 (2)
C18—C17—C16—N3	−162.2 (3)	C3—N8—C5—C6	0.5 (4)
N4—C17—C16—C15	−159.6 (3)	N2—C6—C5—N8	−5.0 (4)
C18—C17—C16—C15	20.0 (4)	C7—C6—C5—N8	173.9 (2)
C20—N7—C18—C17	−2.4 (4)	C5—C6—N2—C2	4.6 (4)
N4—C17—C18—N7	5.7 (4)	C7—C6—N2—C2	−174.3 (2)
C16—C17—C18—N7	−174.0 (3)	C6—N2—C2—C3	−0.3 (4)
C7—N1—C11—C10	0.3 (5)	N8—C3—C2—N2	−4.3 (4)
Ag1—N1—C11—C10	177.5 (2)	C1—C3—C2—N2	175.7 (3)

### Hydrogen-bond geometry (Å, °)

**Table:**

*D*—H···*A*	*D*—H	H···*A*	*D*···*A*	*D*—H···*A*
C5—H5a···F3^i^	0.93	2.49	3.398 (3)	167
C13—H13a···F2	0.93	2.43	3.015 (5)	121
C11—H11a···F4^ii^	0.93	2.39	3.132 (4)	137

Symmetry codes: (i) −*x*+1, −*y*+1, −*z*+1;  (ii) *x*+1, *y*+1, *z*.

### Table 2 π–π interactions

**Table:**

Cg	Cg	Cg···Cg (Å)	Slippage angle
Cg1	Cg1^i^	3.610 (2)	20.71
Cg2	Cg2^ii^	3.926 (2)	33.28
Cg3	Cg3^iii^	3.820 (2)	24.69
Cg4	Cg4^iv^	3.898 (1)	25.89

Slippage angle   = Angle Cg(I)-->Cg(J) normal to plane I Cg1,Cg2,Cg3,Cg4 are the centroids of C2-C3-N8-C5-C6-N2 (pyrazinyl) and C17-C18-N7-C20-C21-N4 (pyrazinyl), C7-C8-C9-C10-C11-N1(pyridyl) and C12-C13-C14-C15-C16-N3(pyridyl) respectively. Symmetry Codes: (i):-x+1, -y+1, -z+1; (ii): -x+1, -y+2, -z; (iii):-x+1, -y+2, -z+1; (iv): -x+1, -y+1, -z
